# Diseases of the Brain Due to Diseases of the Ear

**Published:** 1893-12-30

**Authors:** 


### Diseases of the Brain due to Diseases of the Ear.

We doubt whether there is any branch of medicine which has shown more triumphant progress than this. The diagnostic skill of the physician has so rapidly increased, that he is able to detect and localise the cerebal complications of ear disease at an early stage, and as the operative skill of the surgeons has likewise increased, thanks chiefly to the labours of McEwen, Lane, Ballance, and Horsley, patients are often saved who would a few years ago inevitably have died.

The middle ear is a frequent seat of inflammation, either originating there or spreading up the Eustachian tube. While this is acute complications do not commonly arise, but if it has become chronic, the lining membrane is pervious to micro-organisms, and acute infective inflammation spreads along the lymphatics or veins to the lateral sinus, to the meninges, or to the brain; in any case the results are almost certainly fatal if the patient be left alone. Pus is so confined in the middle ear, that all sorts of micro-organisms flourish luxuriantly in it; it is, in fact, a model incubating chamber, well removed from the influence of the external air, and kept at a uniform temperature, hence the frequency of the above mentioned complications of inflammation of it.

When the antrum is inflamed, the conditions leading to the retention of pus, the development of micro-organisms in it, and damage to the lining membrane, are more favourable. The same is also true of the mastoid cells. From this it follows that any person who has chronic incurable middle ear disease is in a serious condition, for he is—at any moment—liable to meningitis, or cerebral abcess. If there is chronic disease of the antrum his danger is still greater, for this often gives very few external signs of its existence; but if there is any reason to suspect its presence, the antrum must be cut down upon and thoroughly cleared out.

We will now consider the symptoms and treatment of the cerebral complications of middle-ear disease.

*Abscess of the Brain.*—The patient has often had discharge from one of his ears for a long time, but this is by no means always the case, for chronic inflammation of the middle-ear does not always lead to perforation of the membrana tympani. If he has had discharge it may, as he becomes ill, cease. He usually first complains of pain about the ear, which soon extends into the temporal bone, and later it radiates to the frontal and occipital regions. Cerebral vomiting is commonly present, and rigors are a most constant early symptom. The temperature is very little, if at all, raised, and the patient seems prostrate. The symptoms may be difficult to distinguish from those of suppurative middle ear disease, but in a few days the patient passes into a condition characteristic of cerebral abscess. The pain becomes distinctly less, but there may be some tenderness on percussion. Slow cerebration is very evident, and there is a marked want of sustained attention. This mental dulness gradually increases until it becomes very difficult to rouse the patient. In fact, as McEwen well puts it, the mental condition is very like that of opium poisoning. The temperature is still usually not above normal, it may even be sub-normal. The pulse is slow —it may be no more than 30 or 40—and full. The respirations are also slow, and if the abscess is in the cerebellum they may be irregular and of a Cheyne-Stokes character. The bowels are usually constipated, and there is often incontinence of urine. Anorexia is commonly present, but vomiting has usually stopped by now. Paralysis is not often met with, but there may be paralysis of the third nerve from pressure of an abscess in the temporo-sphenoidal lobe or paresis of the opposite side of the body may be present. These symptoms are of great localising value. Sometimes inequality of, or loss of reflex in, one of the pupils is observed. The breath is often foul, the tongue furred, and the teeth covered with sordes. Rigors are now few, or altogether absent. The patient begins to waste, and this, taken with the other signs, greatly helps the diagnosis of cerebral abcess. Optic neuritis is frequent, but usually slight. In one case, recorded by McEwen, an abscess of the temporo-sphenoidal lobe made a minute perforation in the roof of the tympanum, into which drops of pus could be seen to fall. In the final stage death either takes place owing to the gradual deepening of the coma, or to the bursting of the abcess. Cerebral abscess due to middle-ear disease is nearly always in the temporo-sphenoidal lobe; if not there it is in the cerebellum.

*Infective Thrombosis of the Lateral Sinus.*—In ear disease this is probably due to the thrombosis of some small vein in the ear, up which the thrombotic process extends until it reaches the lateral sinus. As the thrombosis is infective the clot soon becomes puriform, and micro-organisms rapidly develope in it, and are very liable to infect the whole body. Not infrequently the thrombosis extends well down into the jugular vein. In very rare cases the spread of the process along the vein which passes through the mastoid foramen leads to the formation of an extra-cranial abcess, or by thrombotic spreading through the posterior condyloid foramen an abcess may form in the posterior triangle. Not uncommonly the infective thrombosis markedly stains and erodes the bone underneath it. There is no tendency to softening of, or hæmorrhage into the brain. The chief symptoms of infective thrombosis of the lateral sinus are as follows: Intense headache usually referred to the seat of the disease; the pain may be excruciating, vomiting of the cerebral type is usually present. The temperature quickly rises to a considerable height, but is subject to marked depressions. The pulse is rapid, small, and thready. Rigors are nearly always present, and often repeated, and followed by profuse perspiration. The breath is foul, the tongue is dry and furred, the bowels are at first constipated, but diarrhœa soon sets in. The respirations are always rapid, and if, as often happens, the lungs are affected secondarily, the patient complains of pain in the chest, and his expectoration is of a prune-juice colour, and may smell horribly. Towards the end of the case he sinks into a typhoid condition, the symptoms of which are well known. If the internal jugular is thrombosed it is felt like a thick cord lying outside the carotid artery. Sometimes the cervical glands are enlarged, and an abcess in the neck may form.

